# Metabolomic strategies and biochemical analysis of the effect of processed *Rehmanniae radix* extract on a blood-deficient rat model

**DOI:** 10.1186/s12906-022-03560-x

**Published:** 2022-03-25

**Authors:** Yang-yang Wang, Ning Zhou, Zeng-fu Shan, Ying-ying Ke, Zhen Liu, Zhen-hui Liu, Wei-sheng Feng, Xiao-ke Zheng

**Affiliations:** 1 College of Pharmacy, Henan University of Chinese Medicine, Zhengzhou, 450046 China; 2 The Engineering and Technology Center for Chinese Medicine Development of Henan Province, 156 Jinshui East Road, Zhengzhou, 450046 China; 3 Key Laboratory of Basic and Application Research of Beiyao, Ministry of Education, Heilongjiang University of Chinese Medicine, Harbin, 150040 China

**Keywords:** Metabolomics, Blood deficiency model, *Rehmanniae Radix*, UPLC-Q-TOF/MS

## Abstract

**Background:**

*Rehmanniae Radix* (RR), an herb with numerous pharmacological effects, is widely used in traditional Chinese medicine for the treatment of blood deficiency syndrome, either alone or in combination with other herbs. However, the mechanism by which processed *Rehmanniae Radix* (PRR) improves blood enrichment efficacy has not been clearly defined.

**Methods:**

Ultra-performance liquid chromatography coupled to quadrupole time-of-flight mass (UPLC-Q-TOF/MS) and biochemical methods were combined to explore the hematopoietic functional mechanisms of PRR on blood deficiency in a rat model, as well as the potential active ingredient for blood enrichment efficacy. The pharmacological effects of PRR were evaluated on a rat blood deficiency model induced by cyclophosphamide in combination with 1-acetyl-2-phenylhydrazine. The blood routine index, including white blood cell (WBC), red blood cell (RBC), and platelet (PLT) counts, as well as hemoglobin (HGB) level, and the changing metabolite profile based on urine and serum were assessed. Nontargeted metabolomic studies, combined with biochemical analyses, were employed to clarify pharmacological mechanisms.

**Results:**

PRR significantly increased the blood routine index levels and reversed the levels of SOD, GSH, and ATP. The PRR group was similar to the control group, as determined from the metabolic profile. All of the 60 biomarkers, representing the typical metabolic characteristics of the blood-deficient rat model, mainly involved energy metabolism dysfunction, the peripheral circulation system, and oxidative damage in the body. This improvement may be attributed to changes in polysaccharide and sixteen non-polysaccharide compounds in PRR, which were caused by processing RR with rice wine.

**Conclusions:**

The strategies of integrated metabolomic and biochemical analyses were combined, revealing the biological function and effective mechanism of PRR.

**Supplementary Information:**

The online version contains supplementary material available at 10.1186/s12906-022-03560-x.

## Background

Blood deficiency is a common syndrome in clinical medicine, which is caused by massive blood loss, nutritional deficiencies, insufficient hematogenesis, and iron deficiency [1]. In modern medicine, it is defined as the reduction of hemoglobin concentration and blood pancytopenia, and it describes a pathological state of blood dysfunction and organ dystrophy in traditional Chinese medicine (TCM) [2, 3]. Acetyl phenylhydrazine (APH), which acts as a strong oxidant, could slowly cause oxidative damage to red blood cells and, ultimately, hemolytic anemia of the body [4]. At present, chemotherapy is the most common clinical treatment of cancer. Cyclophosphamide (CTX), a broad-spectrum anticarcinogen, has strong cytotoxicity for hematinic stem cells in the bone marrow and circulating peripheral blood cells, thereby resulting in anemia (inhibition of hematopoietic function) and immunodeficiency [5]. Thus, there is an urgent needed to identify a drug that can ameliorate blood deficiency caused by chemotherapeutics. In a recent study, a hemolytic and aplastic anemia model induced by APH and combined with CTX has been shown to be quite consistent with the in vivo environment of blood deficiency syndrome in clinical settings [6].

*Rehmanniae Radix* (Dihuang, RR), derived from the root of the perennial plant *Rehmannia glutinosa* (Gaertn.) DC, has been used clinically in TCM for decades. It is classified as a safe medicine, according to *Shennong’s Classic Materia Medica* (Shennong Bencao Jing). It is sweet, edible, and nontoxic [7]. There are two types of RR commonly used in clinical practice, including dried *Rehmanniae Radix* (Sheng Dihuang, DRR) and processed *Rehmanniae Radix* (Shu Dihuang, PRR), which is obtained by sun-drying the fresh root of RR and steaming it with rice wine by traditional methods [8, 9]. In addition, a recent study has shown that the hematopoietic function of PRR was not affected by different processing methods [10]. After processing with rice wine, the supplement blood and reinforced marrow function of PRR was enhanced in TCM [11, 12]. Modern pharmacological studies have shown that PRR exhibits a wide range of actions on the blood system, immune system, endocrine system, cardiovascular system, and nervous system [13].

In this study, an APH and CTX-induced blood deficiency model was established to simulate the pathological state of blood-deficient patients. Then, significant physiological indexes that reflect the status of blood deficiency, including WBC, RBC, and PLT, were measured to assess the treatment effects of PRR. In addition, metabolomic methods were used to evaluate the metabolic profile, biomarkers selection, and metabolic network. The results revealed the metabolic disorders in the blood deficiency model concerning the intervention of PRR [14]. Finally, the results of physiological indexes analysis, difference-compound analysis, and the metabolomic data were integrated to clarify the underlying mechanisms of PRR on the blood deficiency model, as well as the potential active ingredient for blood enrichment efficacy.

## Materials and methods

### Chemicals and reagents

1-Acetyl-2-phenylhydrazine (APH) was purchased from Shanghai Aladdin industrial Co., Ltd. (batch number: 201406, Shanghai, China). Cyclophosphamide (CTX) was purchased from Jiangsu Hengrui Medicine Co., Ltd. (batch number: 18062625). Positive control drug, Asini Corii Colla (ACC), was purchased from Shandong Donge Co., Ltd. (batch number: 1709003).

The levels of SOD (A001–1-2, Nanjing Jiancheng Bioengineering Institute, China), GSH (C011–2, Suzhou Kaerwen Bioengineering Institute), GSH-Px (C006–2-1, Nanjing Jiancheng Bioengineering Institute), and MDA (C003–1-2, Suzhou Kaerwen Bioengineering Institute) in serum and ATP (202,005, Suzhou Kaerwen Bioengineering Institute) in the liver were measured using specific kits, in accordance with the manufacturer’s instructions. All assays were performed in triplicate.

Chromatographic -grade methanol and acetonitrile were purchased from Fisher Scientific (Bridgewater, NJ, USA). Ultrapure water was produced by a Milli-Q water purification system (Millipore, Bedford, MA, USA), and formic acid (LC/MS grade) was purchased from Fisher Scientific (Bridgewater, NJ, USA).

#### Preparation of extracts and polysaccharide samples

DRR was collected in Wuzhi County, Henan Province, China, and was identified by professors Chengming Dong and Suiqing Chen of Henan University of Chinese Medicine. We received approval for sampling in line with the regulations of Peraturan project 2017YFC1702800. The voucher specimen was deposited in a material room at pharmaceutical chemistry of Henan University of Chinese Medicine by code 20171120A. To prepare the PRR extract, the traditional method of processing by steaming rice wine was employed. Briefly, 2000 g DRR was mixed with 1000 mL rice wine in an airtight space for 48 h to achieve homogeneous softness. The mixture was steamed for 24 h over boiling water and then dried at 55 °C to generate PRR. The DRR and PRR were extracted three times with boiling water (1:10) for 1 h, and were filtered through gauze. Then, the merged mixtures were condensed under decompression. Finally, the DRR and PRR extracts were made to a concentration of 1 g crude extract/mL.

DRR and PRR polysaccharide and non-polysaccharide samples were obtained from the abovementioned extraction solution using 80% ethanol for precipitation at 4 °C for 24 h. Then, the samples were centrifuged at 6000 rpm for 10 min. The precipitate was washed three times with 80% ethanol before use in a polysaccharide assay for the phenol sulfuric acid colorimetry method. The supernatant was used for non-polysaccharide multivariate analysis by UPLC-Q-TOF/MS.

### Animal handling

SPF female Sprague–Dawley rats (weighing 180–220 g) were provided by Beijing Vital River Laboratory Animal Technology Co., Ltd. (animal license number: SCXK (Jing) 2016–0006; Beijing, China). The rats were housed in constant conditions, with room temperature of 20–25 °C, relative humidity of 55–65%, and a 12 h light/dark cycle. All of the animals were free to access food and water. All of the animal procedures were performed in accordance with the Guidelines for Care and Use of Laboratory Animals of the Henan University of Chinese Medicine, and the experiments were approved by the Animal Ethics Committee of Henan University of Chinese Medicine (DWLL201903052). The study was carried out in compliance with the ARRIVE guidelines.

After one-week acclimatization, 40 rats were randomly divided into the following four equal groups: the control group (C); the blood deficiency model group (M); the Asini Corii Colla positive group (ACC, as a positive control group); and the processed PRR treatment group. In the morning, the rats of the M, ACC, and PRR groups were hypodermically injected with 2% APH saline solution at a dose of 20 mg·kg^− 1^ on day 1, and at a dose of 10 mg·kg^− 1^ on day 4. Two hours after the injection of 2% APH saline solution on day 4, the rats were intraperitoneally injected with CTX saline solution on days 4, 5, 6, and 7 at a dose of 20 mg·kg^− 1^. The control group received an equal volume of saline [15]. In the afternoon, the rats of the ACC and PRR groups were given ACC (3 g·kg^− 1^) and PRR extract (7 g·kg^− 1^, equal to 7 mL·kg^− 1^ body weight) intragastrically in line with the commonly used doses of the Chinese Pharmacopoeia and literature reports [15]. The drugs were administered one time each day for 15 continuous days. The C group and M group were given water over the 15-day period.

### Biochemical assessment

The levels of SOD, GSH, MDA, and ATP in the liver were measured using ELISA kits in accordance with the manufacturer’s instructions (Nanjing Jiancheng Bioengineering Institute and Suzhou Kaerwen Bioengineering Institute, China). The peripheral hemogram index was determined using an Auto Hematology Analyzer (BC-2800 Vet, Mindray Animal Medical Technology Co.).

### Sample collection and preparation

Blood samples were collected via the abdominal aorta on day 15. One part was collected in evacuated tubes with EDTA-K2 for the determination of white blood cell (WBC), red blood cell (RBC), and platelet (PLT) counts, as well as hemoglobin (HGB) level. The other part was allowed to clot at room temperature for 1 h and then centrifuged at 3000 rpm for 10 min. Then, the supernatants (serum samples) were separated and stored at − 80 °C until analysis. A urine sample was collected by metabolic cage at 12 h after the administration. All urine samples were immediately centrifuged at 12000 rpm for 10 min at 4 °C. Then, the supernatants were collected and stored at − 80 °C until further analysis.

Prior to analysis, serum and urine were thawed at room temperature and then 600 μL of cold acetonitrile was added to 200 μL of serum to precipitate proteins. After vortexing for 1 min and incubation on ice for 10 min, the mixture was centrifuged at 12000 rpm for 10 min at 4 °C. To a 300 μL urine sample, 900 μL cold acetonitrile was added to precipitate proteins. The mixture was vortexed for 1 min and centrifuged at 12000 rpm for 10 min at 4 °C. The quality control (QC) samples were obtained from aliquots of the whole sample set. The supernatants of the serum and urine samples were transferred to auto-sampler vials, and 2 μL of each sample was injected into the UPLC system.

### Metabolic profiling analysis of serum and urine

Chromatographic experiments were performed on an Acclaim™ RSLC 120 C18 column (2.1 × 100 mm, 2.2 μm) using a Dionex UltiMate 3000 system (Thermo Scientific, USA). The column temperature was maintained at 40 °C, and the mobile phase consisted of water (containing 0.1% formic acid, A) and acetonitrile (B), at a flow rate of 0.3 mL/min. For separation of serum samples, the gradient elution program was as follows: 0–2 min, 10–60% B; 2–4 min, 60–70% B; 4–5 min, 70–75% B; 5–13 min, 75–77% B; 13–19 min, 77–80% B; 19–20 min, 80–90% B. For separation of urine samples, the gradient elution program was as follows: 0–1 min, 5–15% B; 1–10 min, 15–18% B; 10–13 min, 18–75% B; 13–19 min, 75–85% B; 19–20 min, 85–95% B.

Mass spectrometry was performed on maXis HD QTOF-MS system (Bruker, Germany) with an ESI source. The capillary voltage was 3500 V and 3200 V in the positive and negative ion modes, respectively. The nebulizer pressure was 2.0 bar, and the dry gas temperature and flow rate were 230 °C and 8 L·min^− 1^, respectively. The quality control (QC) sample was injected six consecutive times at the beginning of the sample sequence to condition and equilibrate the system. The QC sample was also injected after every 10 samples throughout the run to further monitor the stability of the analysis.

### Data processing and statistical analysis

The raw mass data were normalized and recalibrated by Profile Analysis (version 2.1, Bruker Germany). The background noise was subtracted; peak alignment was performed; and the generated bucket table was introduced to SIMCA (version 14.1, Umetrics AB, Sweden) software for multivariate statistical analysis. Principal component analysis (PCA) was performed as the unsupervised method for outlier identification and data visualization, and orthogonal partial least squares discriminant analysis (OPLS-DA) was performed for metabolic profiling visualization and biomarker identification. A potential biomarker was selected by variable importance for the projection (VIP) value (VIP > 2.5) and the *t* test (*P* < 0.05, SPSS software version 19.0). Biomarkers were identified by available biochemical databases, such as the Human Metabolome Database (HMDB) (http://www.hmdb.ca/) and KEGG (http://www.genome.jp/kegg/), based on the accurate mass and tandem mass spectrometry (MS/MS) fragments. The cross-validation parameter R^2^ and Q^2^ values are important indicators of explained variation and model predictability, respectively. Furthermore, heat maps were constructed using MEV software (version 4.9.0). Pathway analysis and network construction for the affected pathway were performed via MetaboAnalyst (http://www.metaboanalyst.ca/) and KEGG (http://www.kegg.jp/).

## Results

### Physical characteristics and biochemical parameters

**Fig. 1:**
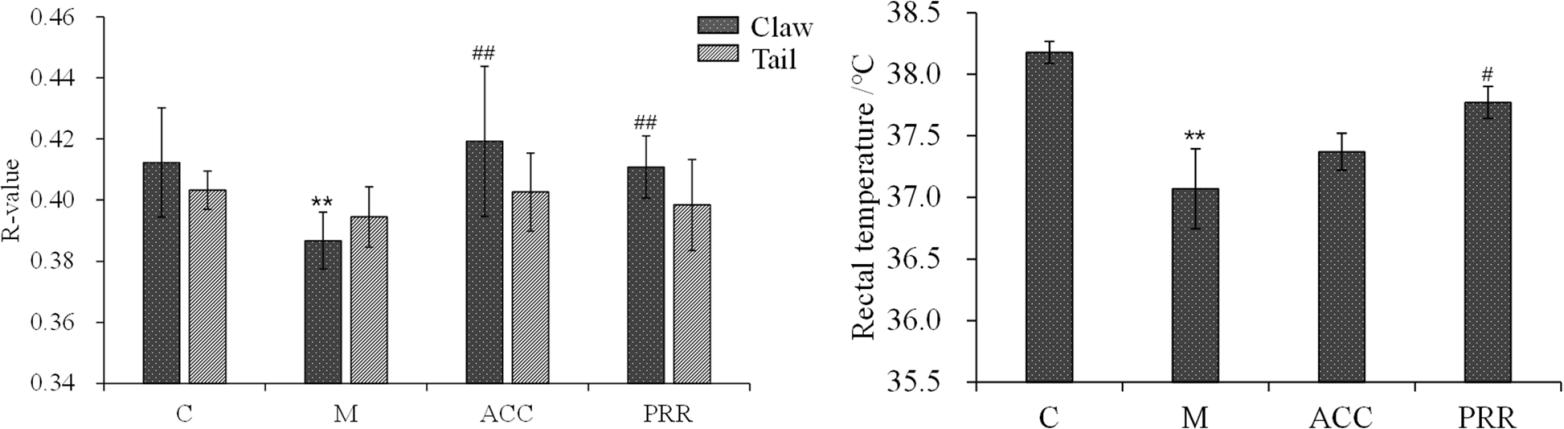
The effects of PRR decoction extract on the *R*-value and rectal temperature of blood-deficient rats. #*P* < 0.05, ##*P* < 0.01 compared with the control group. *P < 0.05, **P < 0.01 compared with the model group

On day 15, the blood deficiency model group appeared exhausted, sluggish, and lethargic. The tail, ears, and eyes were pale, and the body was cool. RGB is an international standard of quantification, and the red color channel can be used to evaluate the redness of the tail and claw in animals [16, 17]. As shown in Fig. 1, the R-value and body temperature of the model group decreased significantly, suggesting that the physical characteristics were consistent with blood deficiency symptoms. However, the ACC and PRR groups showed improved physical characteristics. The WBC, RBC, and PLT counts, as well as HGB and HCT levels, were reduced in the model groups (Table 1). For the blood deficiency model, PRR significantly improved the peripheral hemogram. This is consistent with a previous study, which showed that PRR had hematopoietic effects and that DRR did not [8]. This result indicates that the blood enrichment effect of DRR was enhanced after rice wine processing.

**Table 1:** The effects of PRR decoction extract on the peripheral hemogram of blood deficiency rats

Group	RBC / × 10^12^/L	WBC / × 10^9^/L	HGB / g·L^− 1^	PLT / ×10^9^/L	HCT /%
C	6.97 ± 0.53	11.97 ± 1.20	133.9 ± 10.5	1216.6 ± 83.1	42.77 ± 3.28
M	3.46 ± 0.28##	4.68 ± 1.13##	82.1 ± 6.9##	626.5 ± 61.2##	30.47 ± 2.51##
ACC	4.90 ± 0.43**	6.94 ± 0.92**	109.4 ± 4.4**	908.9 ± 51.8**	34.89 ± 2.51**
PRR	6.16 ± 0.27**	9.80 ± 1.54**	124.8 ± 6.0**	1193.3 ± 65.5**	39.63 ± 1.98**

#*P* < 0.05, ##*P* < 0.01 compared with the control group. *P < 0.05, **P < 0.01 compared with the model group

### Metabolic profile of serum and urine samples

**Fig. 2:**
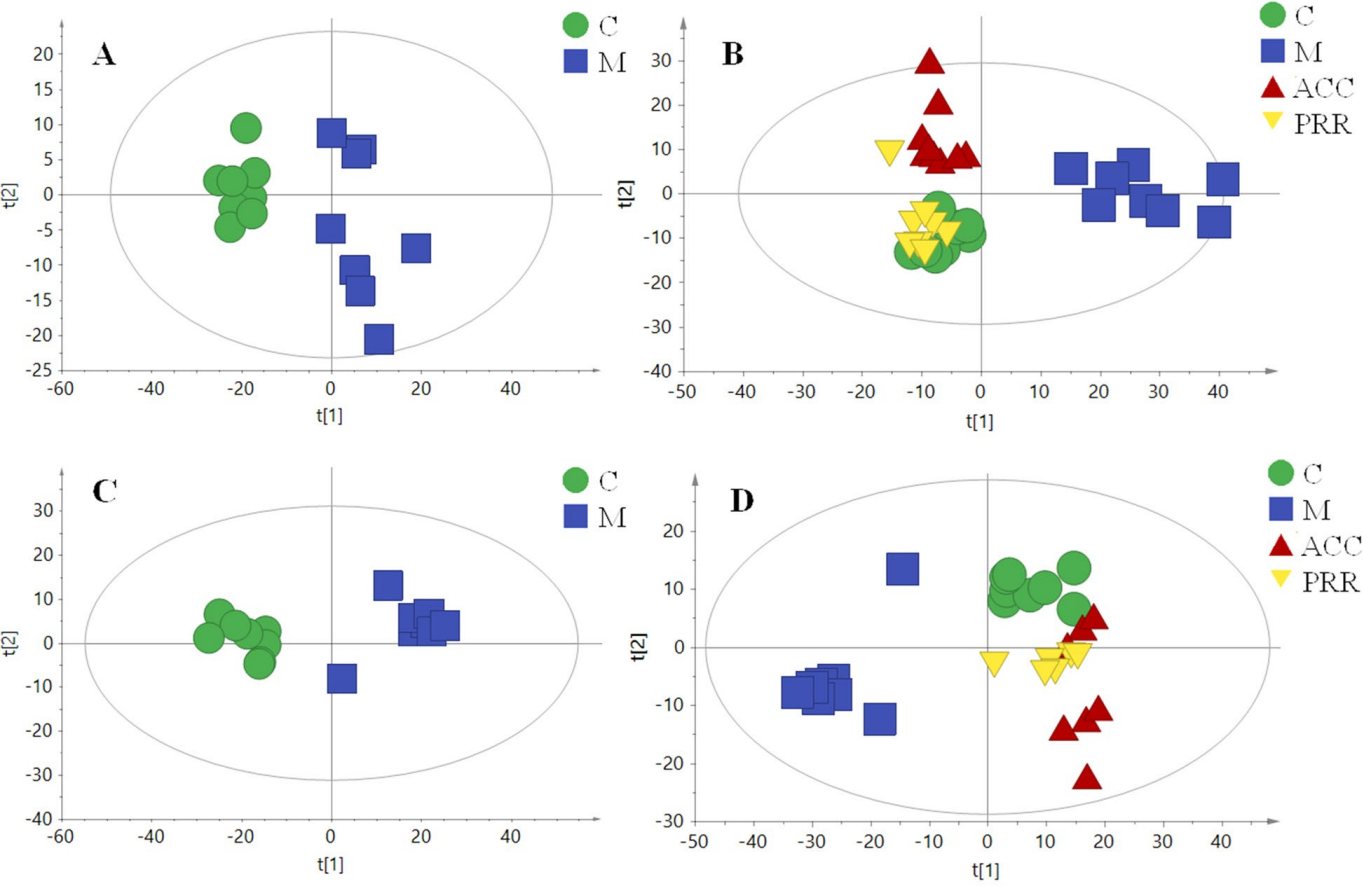
The PCA score scatter plots of the effect of ACC and PRR decoction extracts on blood-deficient rats. The PCA plot of serum (**A** and **B**) and urine samples (**C** and **D**)

The PCA score plots of serum and urine were generated to evaluate the effects of PRR on blood-deficient rats. In the metabolic profile analysis, the number of all serum samples in the PCA score scatter plot was greater than 8 (Fig. 2 A and B), and the number of all urine samples in the PCA score scatter plot was greater than 6 (Fig. 2 C and D). As a result (Fig. 2 A and C), the control group was separated from the model group, indicating that the metabolic profile changed significantly for the model group. The ACC and PRR groups were all clustered with the C group (Fig. 2 B and D), suggesting that ACC and PRR exhibited a similar effect on blood-deficient rats, and this influence may be attributed to the ability of the drugs to restore normal metabolic status to some degree. This result was consistent with the biochemistry results.

#### Potential biomarkers

**Fig. 3:**
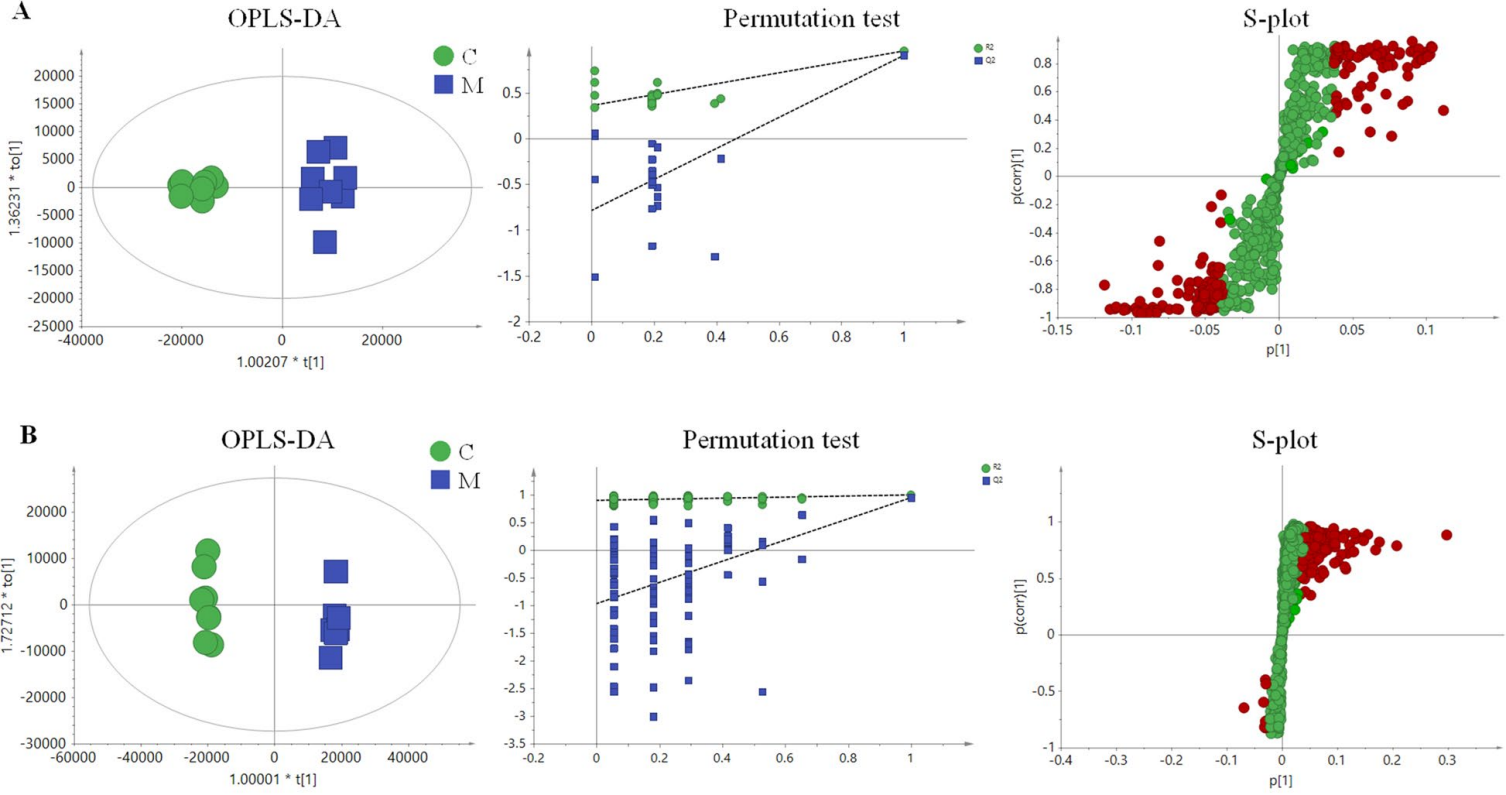
The OPLS-DA score scatter plots of the C group and the M group, 300-iteration permutation test, and the OPLS-DA plot of serum (**A**) and urine (**B**) samples

To elucidate more detailed metabolic differences between the C group and the M group, supervised regression modeling (OPLS-DA) of serum was conducted. As shown in Fig. 3 A, the C and M groups were clearly separated. A 300-iteration permutation test indicated that the values of permuted R^2^ and Q^2^ (bottom left) were significantly lower than the original R^2^ and Q^2^ values (top right), suggesting that the OPLS-DA models provided good prediction. In addition, the same strategies were applied on the urine samples. As shown in Fig. 3B, the C and M groups were clearly separated, suggesting that the urine sample metabolic profiles of blood-deficient rats were altered by CTX and APH induction. A 300-iteration permutation test (Fig. 3 B) showed a result similar to that of the serum sample, indicating good prediction. Before consideration as endogenous biomarkers, these metabolites were carefully filtered using the *t* test (*P* < 0.05) and VIP value (VIP > 3.0). The metabolites were identified by searching online databases, such as the HMDB and KEGG, based on the information of accurate mass of tandem mass spectrometry (MS/MS) fragments. A total of 40 potential biomarkers in serum and 20 potential biomarkers in urine related to blood deficiency were identified (supplementary Table 1).

**Fig. 4:**
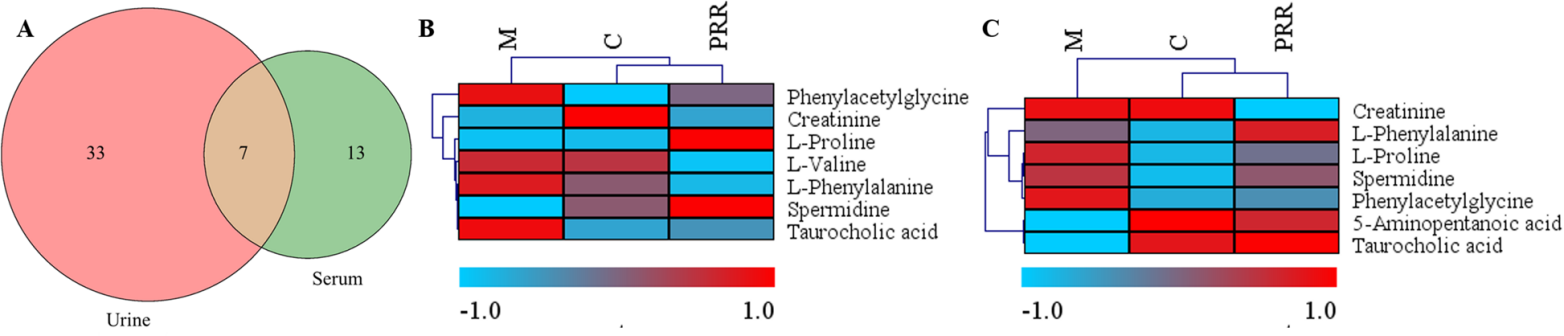
The Venn diagram between urine and serum biomarkers (**A**) and cluster analysis of the seven common biomarkers in urine (**B**) and serum (**C**)

**Fig. 5:**
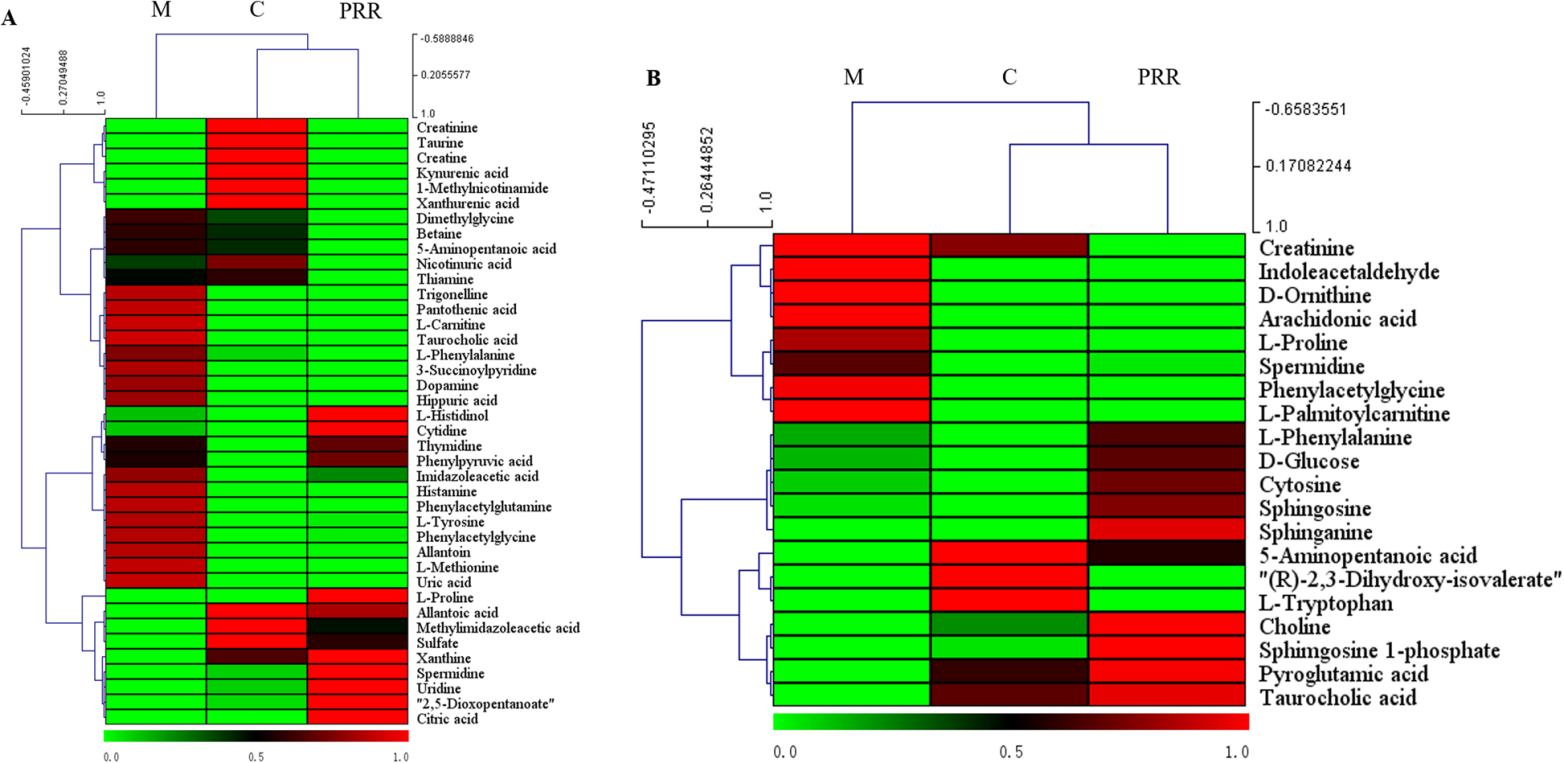
The heat map cluster analysis of 40 potential biomarkers in urine (**A**) and 20 potential biomarkers in serum (**B**). A color change from green to red indicates the level of increased extent

Seven biomarkers were identified in both serum and urine (Fig. 4 A). The cluster analysis results (Fig. 4 B and C) showed that the PRR group closely clustered with the control group with seven biomarkers, suggesting that this could represent the cluster result-based serum and urine sample. Additionally, heat map cluster analysis was used to view the variation in metabolite levels among different groups. As shown in Fig. 5, the PRR group for urine and serum closely clustered with the control group, indicating that PRR could ameliorate the metabolite levels of blood-deficient rats. PRR clearly exhibited hematopoietic effects through the adjustment of the metabolite levels.

### Correlation analysis of significant biomarkers in urine and serum

**Fig. 6:**
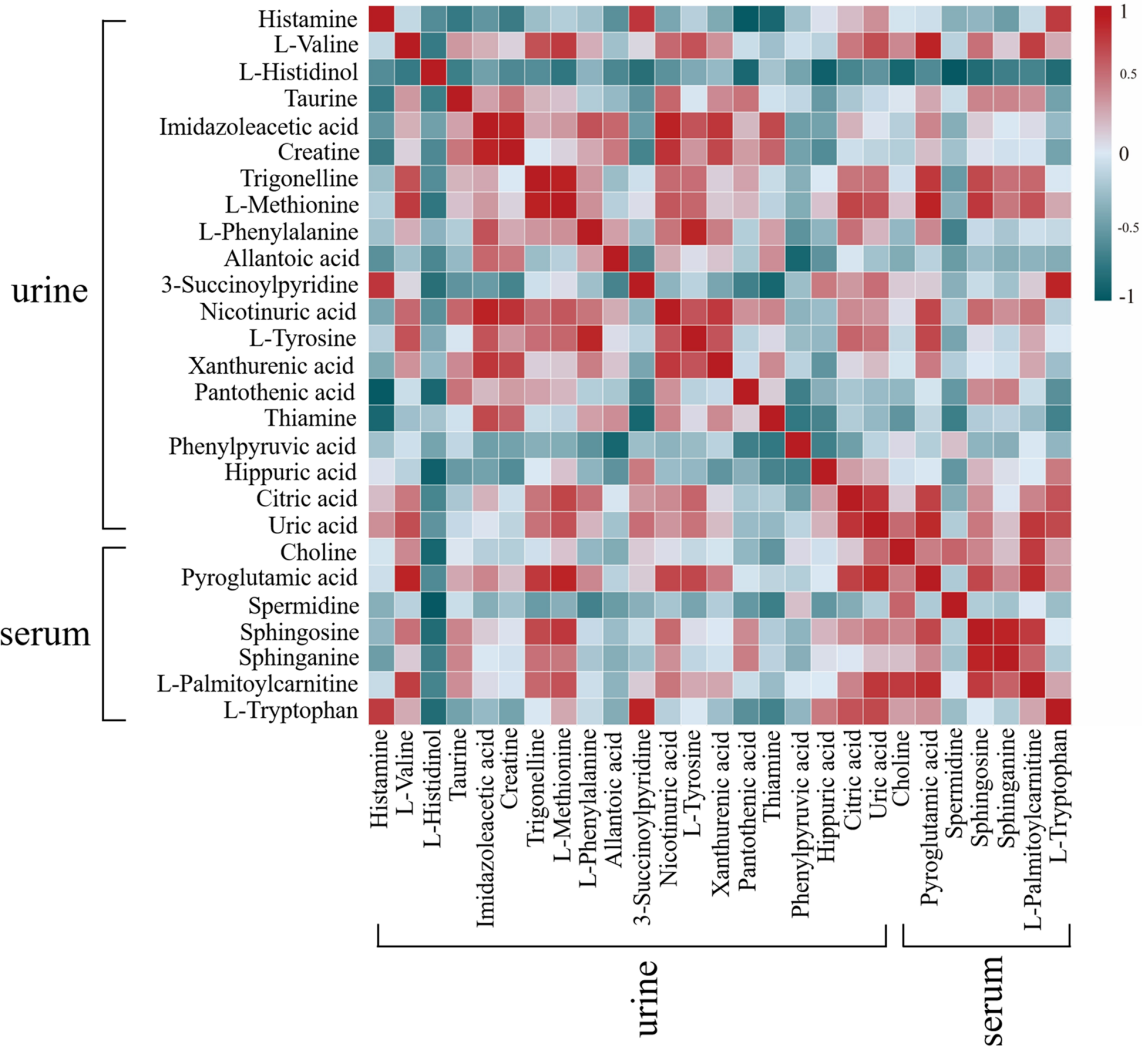
Correlation analysis of key metabolites in the PRR groups of urine and serum on day 15. The correlation degrees are shown using a color scale from a significantly negative correlation (blue) to a significantly positive correlation (red)

Both the peripheral hemogram index and metabolite profile suggested that PRR showed a significant blood-enriching effect, which could be attributed to the changed metabolites after the PRR administration. The mutual metabolite correlations within the PRR group of urine and serum were calculated (Fig. 6). Those metabolites were associated with energy metabolism (L-valine, betaine, choline, citric acid), peripheral circulation system metabolism (histamine, nicotinuric acid, hippuric acid), and redox metabolism (taurine). The biomarkers of the metabolites showed a positive correlation, meaning that the PRR could positively adjust the corresponding pathways to enrich blood.

### Network analysis of affected pathways

**Fig. 7:**
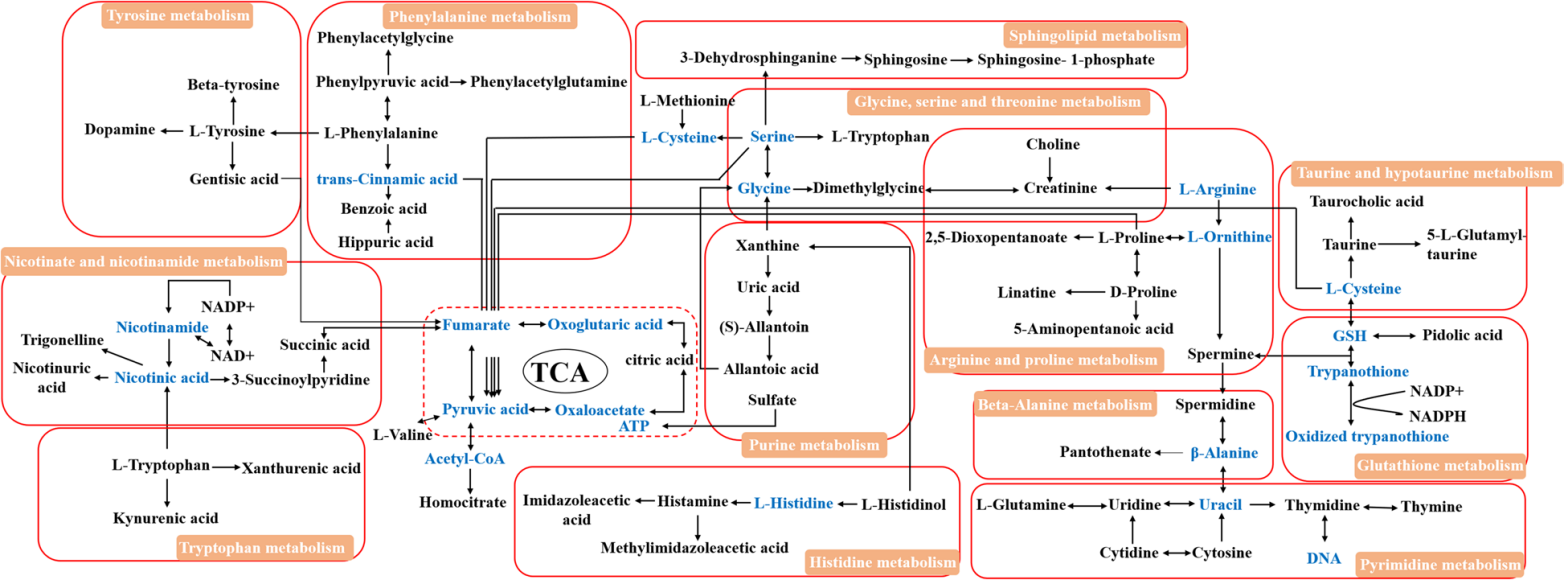
The metabolic network response to PRR exposure. The dark biomarkers were detected in all treatment groups; the light green biomarkers represent the metabolites in the corresponding pathway

**Fig. 8:**
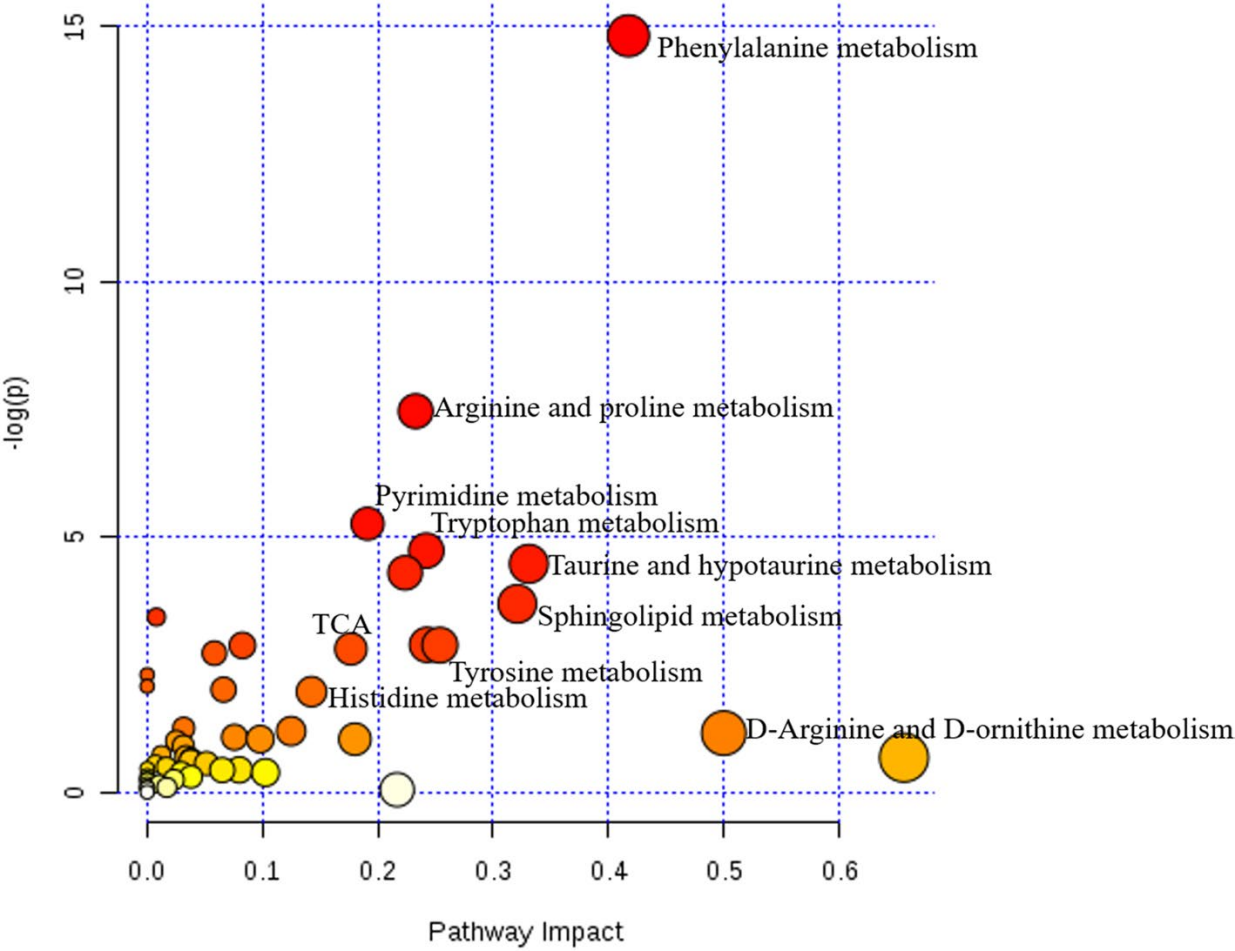
The pathway analysis of blood-deficient rats

The ingenuity network analysis was adopted to explore the relationships among metabolites and metabolite networks in urine and serum. All 60 biomarkers in serum and urine were analyzed by MetPA, which identified the most relevant metabolite pathways by combining the results of pathway enrichment analysis and topology analysis. As shown in Fig. 8, nine metabolic pathways were associated with the host response to blood deficiency, namely, phenylalanine metabolism, arginine and proline metabolism, pyrimidine metabolism, tryptophan metabolism, TCA cycle, taurine and hypotaurine metabolism, sphingolipid metabolism, tyrosine metabolism, and histidine metabolism. According to our results and the information provided by the KEGG database [18–20], a metabolite correlation network of PRR was constructed. As shown in Fig. 7, those metabolite pathways, especially the TCA cycle, were closely interconnected with each other.

**Fig. 9:**
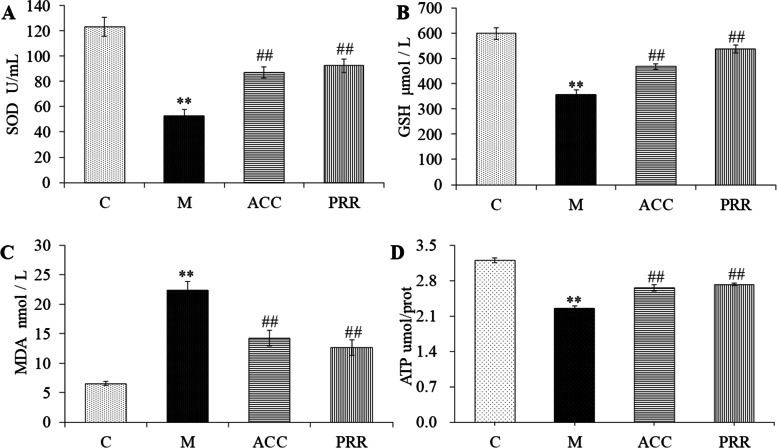
The effect of PRR on oxidative stress and energy metabolism in rats with blood deficiency. The effect of PRR on SOD (**A**), GSH (**B**), MDA (**C**), and ATP (**D**). ** *P* < 0.01 compared with the control group. ## *P* < 0.01 compared with the model group

**Fig. 10:**
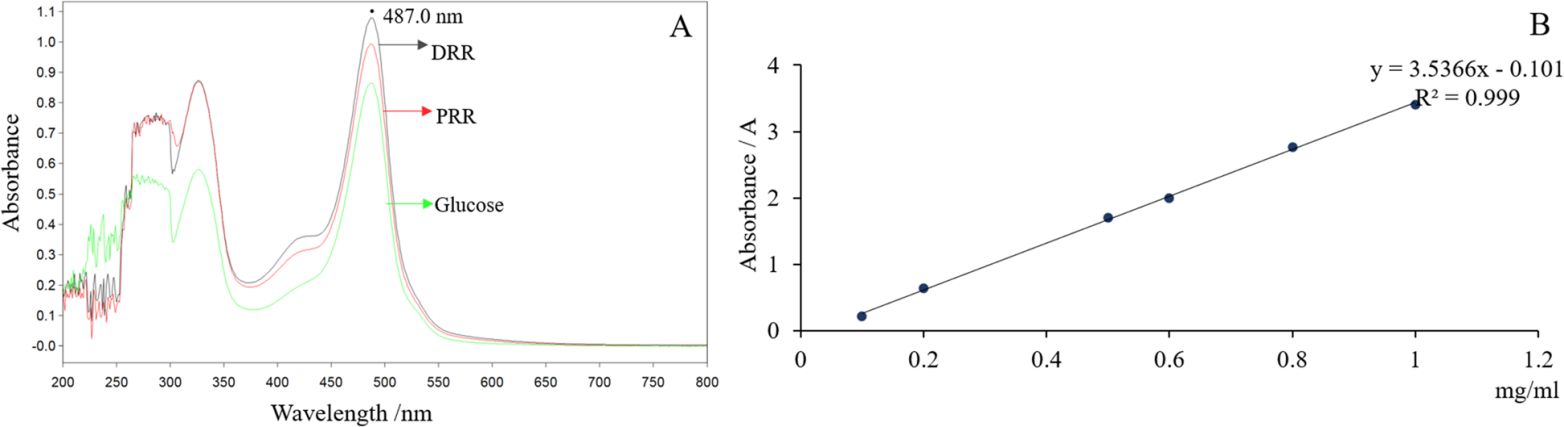
The maximum absorbance value at different concentrations (**A**); the standard curve (**B**)

**Fig. 11:**
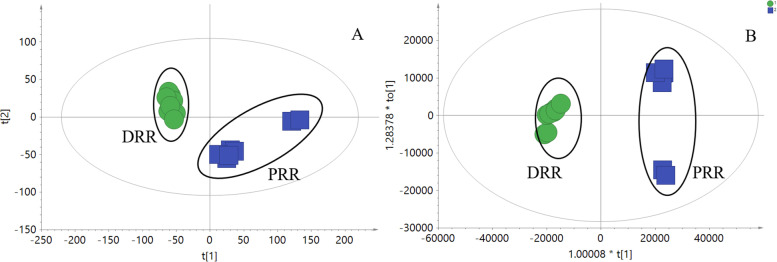
PCA score plot (**A**) and OPLS-DA score plot (**B**) comparison of DRR and PRR extracts through UPLC-Q-TOF/MS detection

### Verification of oxidative stress and energy metabolism state in the blood deficiency rat model

The biomarker and pathway analyses of blood-deficient rats mainly referred to energy metabolism dysfunction, peripheral circulation system, and oxidative damage in the body. To further validate the regulatory effect of PRR on energy metabolism and oxidative damage in the body, the activities of key enzyme were analyzed by ELISA kits. The results (Fig. 9) revealed that the levels of SOD, GSH, and ATP significantly decreased in rats with blood deficiency. In contrast, the enzyme levels increased after PRR treatment. Additionally, the levels of MDA were reversed by PRR (Fig. 9C). Decreased levels of ATP (Fig. 9D) suggested that the energy metabolism was slowed down in the model group, which could reduce the ability to scavenge free radicals and result in lipid peroxidation of the cell membrane, reduced SOD activity, and reduced stability of the cell membrane. Meanwhile, the state and the metabolic mechanism of the biomarkers and pathways identified by metabolomic were verified.

### The compositional differences between DRR and PRR extracts

Finally, to explore the potential component of PRR responsible for the blood enrichment effect, this study analyzed the difference in polysaccharide content and non-polysaccharide composition between DRR and PRR extracts. As shown in Fig. 10, polysaccharide has a maximum absorbance at 487 nm, and the absorbance values of DRR and PRR were 0.401 and 0.810, respectively. With concentration as the x-axis and absorbance as the y-axis, the regression equation y = 3.5366 x − 0.101 (*R*^2^ = 0.999) was calculated. The absorbance was linear from 0.1 mg·mL^− 1^ to 1 mg·mL^− 1^, with an *R*^2^ = 0.999. Using the regression equation, the content of polysaccharide in DRR and PRR was 91.7 and 16.36 mg·g^− 1^, respectively. This suggests that polysaccharide is one of the components that may be responsible for the potential difference in blood enrichment efficacy.

Then, the non-polysaccharide composition was analyzed via UPLC-Q-TOF/MS and multivariate analysis in SIMCA software. The results are shown in Fig. 11. The PCA and OPLS-DA scores were significantly separated, indicating that the DRR composition changed after rice wine processing. A total of 16 different compounds were identified using standards separated by our team before the study (Table 2).

**Table 2:** Compounds differing between DRR and PRR extracts, based on multivariate analysis

No.	Compound	Formula	Calculated (Da)	Detected (Da)	Error (ppm)	Related changed trend
1	5-hydroxymethyl furaldehyde	C_6_H_6_O_3_	149.0209	149.0209	0.00	↑
2	Dihydrocatalpol	C_15_H_24_O_10_	387.1262	387.1259	−0.77	↓
3	Adenosine	C_10_H_13_N_5_O_4_	268.1040	268.1035	−1.86	↓
4	Melittoside	C_21_H_32_O_15_	547.1633	547.1633	0.00	↓
5	Rehmannioside D	C_27_H_42_O_20_	709.2161	709.2152	−1.27	↓
6	Guaicylglycerol	C_10_H_14_O_5_	237.0733	237.0753	−2.11	**+**
7	Rehmannia glutinin A	C_19_H_34_O_8_	413.2146	413.2140	−1.45	↓
8	Pterolactam	C_5_H_9_NO_2_	116.0706	116.0706	0.00	↓
9	Harman-3-carboxylic acid	C_13_H_10_N_2_O_2_	227.0815	227.0812	−1.32	↑
10	Echinacoside	C_35_H_46_O_20_	809.2475	809.2470	−0.62	↑
11	Aucubin	C_15_H_22_O_9_	369.1156	369.1153	−0.81	↓
12	Leonuride	C_15_H_24_O_9_	371.1312	371.1308	−1.08	–
13	Rehmannia glutinin C	C_19_H_32_O_7_	373.2221	373.2215	−1.61	↓
14	Catalpinoside	C_15_H_22_O_10_	385.1105	385.1102	−0.78	↓
15	6-*O*-vanilloylajugol	C_23_H_30_O_12_	521.1629	521.1634	0.96	–
16	Rehmannia glycoside	C_31_H_48_O_18_	731.2733	731.2728	−0.68	↓

Note: ↓↑ represent the direction of changes in content after processing (PRR); **+**, **−** represent newly generated and absent compounds after rice wine processing (PRR sample)

## Discussion

### Energy metabolism

Biosynthetically, valine, leucine, and isoleucine are produced from pyruvate, and are degraded to acetoacetate and acetyl-CoA with the enzyme affected, which involves energy metabolism [21]. L-valine is a branched-chain essential amino acid that acts as an intermediary metabolite, forming succinyl-CoA in the TCA cycle. In this study, (R)-2,3-dihydroxy-isovalerate and its downstream metabolite L-valine were detected in serum and urine. The decrease in (R)-2,3-dihydroxy-isovalerate concentration and the increase in L-valine concentration were attributed to the inhibited degradation of L-valine in the model group. This resulted in limited energy metabolism. After the administration of PRR, these metabolites recovered notably. Therefore, PRR could mitigate blood deficiency via the valine, leucine, and isoleucine biosynthesis pathway.

Glycine, serine, and threonine are glycogenic amino acids. They play key roles in the energy metabolism pathway, which mainly supplies precursors for the TCA cycle [22]. In this study, creatine, betaine, and choline were detected in serum and urine. Betaine and choline can provide methyl groups, thereby supplementing the amount of methyl needed for creatine synthesis. Creatine, an important energy storage compound, is converted into phosphocreatine via creatine kinase. Creatine levels decreased in the model group, indicating that the energy metabolism was inhibited in blood-deficient rats. Choline is an essential element of the cell membrane, and it is related to cell membrane damage [23]. The level of choline decreased in the serum of the blood-deficient group, suggesting that the hematopoietic functions of the cells were impaired. Betaine plays an essential role in adjusting osmoregulatory and oxidative stress, which activates the membrane of damaged red blood cells [24]. As a result, urinary betaine content increased in the blood-deficient model, indicating that APH had induced oxidative stress. After the administration of PRR, the levels of creatine and betaine significantly increased, returning to normal levels. This finding was similar to the peripheral hemogram of blood-deficient rats. In summary, PRR treatment restored the levels of creatine and betaine to improve energy metabolism and balance oxidation–reduction reactions. In addition, PRR could regulate the level of choline and stabilize the cell membrane via glycine, serine, and threonine metabolism.

Citric acid is an intermediate product of TCA. TCA is a common pathway of glucose metabolism, lipid metabolism, and protein metabolism, as well as the main source of energy in vivo [25]. The level of citric acid decreased in the blood-deficient rat model. However, an increase in citric acid level was detected after PRR treatment, suggesting that PRR could mitigate the blood deficiency syndrome by improving energy metabolism.

### Peripheral circulation system

In histidine metabolism, histamine, imidazoleacetic acid, and L-histidinol are generated. As shown in Fig. 7, histamine and imidazoleacetic acid are downstream metabolites of histidine, and they are formed from histidine by histidine decarboxylase. Therefore, a high level of histamine and imidazoleacetic acid in the blood-deficient model indicated a low level of histidine. As recently reported, the chronic lack of histidine could lead to a negative nitrogen balance, reduction of plasma protein and blood volume, and anemia [26, 27]. In this study, the lower level of histidine closely correlated with HGB in the peripheral blood indexes. After PRR treatment, the level of histidine increased, suggesting that blood deficiency syndrome could be mitigated via upregulation of histidine.

Nicotinuric acid, trigonelline, and 3-succinoylpyridine are products of nicotinate and nicotinamide metabolism, which use nicotinamidase and nicotinate N-methyltransferase, respectively. Recently, it has been reported that trigonelline correlates with WBC count [28]. Nicotinamide is a part of coenzymes I and II, and plays an important role in lipid metabolism, respiratory oxidation chain, and anaerobic glycolysis [29, 30]. In this study, these metabolite levels were elevated in the blood-deficient model group. After administering PRR, the metabolites levels decreased, suggesting that PRR could regulate energy metabolism via nicotinate and nicotinamide metabolism.

L-phenylalanine and hippuric acid in phenylalanine metabolism and L-tyrosine in tyrosine metabolism were detected. L-phenylalanine and L-tyrosine are involved in TCA through the forms of fumaric acid. Moreover, they participate in the metabolism of heme and hemoglobin [31]. In addition, the increased levels of L-phenylalanine and L-tyrosine in the model group were closely related to the synthesis of glutathione [32]. After the administration of PRR, these metabolites returned to normal levels, similar to the control group. Correspondingly, this response was attributed to the increase in HGB content. Hippuric acid is excreted in urine [33] and decomposed into pyruvate and acetyl-CoA via a series of biodegradation processes [34, 35]. These metabolites are involved in TCA cycle and affect the synthesis of HGB. The high level of hippuric acid contributed to the inhibition of biodegradation processes in the blood deficiency model, and the content of its metabolite, pyruvic acid, decreased correspondingly. However, the level significantly increased after PRR administration, suggesting that PRR could improve the synthesis of hemoglobin metabolites through the downregulation of hippuric acid.

1-phosphate sphingosine could cause the release of Ca^2+^ from the endoplasmic reticulum and inhibit RBC apoptosis by binding to Ca^2+^ via phospholipid-binding protein-annexin A1 [36, 37]. The level of 1-phosphate sphingosine decreased in the blood deficiency model. After administration of PRR, the sphingosine-1-phosphate levels increased significantly, which promoted the release of Ca^2+^ in the endoplasmic reticulum, inhibited the apoptosis of erythrocytes, and improved the symptoms of blood deficiency.

Arachidonic acid, an important metabolite in arachidonic acid metabolism, is converted to prostaglandins and thromboxane [38]. Arachidonic acid could regulate blood cell function, reduce blood viscosity, and improve physiological activities [39]. Thromboxane A2 (TXA2) is an important mediator, which can accelerate platelet aggregation and promote vasospasm [40]. The level of arachidonic acid increased in the blood deficiency model, and the level of TXA2 increased correspondingly. The levels decreased after PRR administration, suggesting that the peripheral blood routine parameters and function of blood cells improved through the downregulation the arachidonic acid.

### Oxidative damage and stability of cell membrane

Taurine is an important sulfur-containing amino acid that is ubiquitous in the body. It possesses numerous physiological roles. Taurine acts as an osmoregulatory compound to maintain the osmotic pressure and stability of the cell membrane by Ca^2+^, Mg^2+^, Na^+^, and K^+^ plasma ion transport [41, 42]. As an antioxidant, taurine reduces oxidative damage to the cell membrane caused by APH, which induces oxidative damage on RBC resulting in hemolytic anemia [43, 44]. Moreover, taurine can improve energy metabolism in the body [45]. The level of taurine significantly decreased in the blood deficiency model; however, after administering PRR, the level of taurine increased. The decreased SOD and GSH levels and the increased MDA levels in the rat model group with blood deficiency indicated the status of oxidative damage. After the administration of PRR, the levels of SOD, GSH, and MDA were reversed. Additionally, the levels of SOD, GSH, MDA, and ATP were closely related to taurine levels, reflecting the oxidation–reduction and energy metabolism status of the rat model with blood deficiency. Therefore, our results indicate that PRR could regulate taurine and hypotaurine metabolism to improve blood deficiency.

## Conclusion

In this study, an integrated metabolomic approach, utilizing UPLC-Q-TOF-MS coupled with the efficacy index, was applied to explore the hematopoietic functional mechanisms and the potential different components of PRR. In serum and urine, nine metabolic pathways related to blood deficiency, including TCA cycle, phenylalanine metabolism, arginine and proline metabolism, pyrimidine metabolism, tryptophan metabolism, taurine and hypotaurine metabolism, sphingolipid metabolism, tyrosine metabolism, and histidine metabolism, were mitigated by PRR treatment. Therefore, the restorative effect of PRR could be attributed to the potential different components that changed with the processing with rice wine. Combined with the peripheral hemogram index, PRR exhibited desirable effects on blood-deficient rats through the adjustment of energy metabolism, peripheral circulation system, and oxidative damage in the body. Additionally, the polysaccharide content and 16 non-polysaccharide compounds served as indicators of the hematopoietic function. In summary, this integrated metabolomic approach successfully revealed the restorative effects and the underlying mechanisms of PRR, providing a theoretical foundation for clinical application.

## Supplementary Information

**Additional file 1.**

## Data Availability

All of the data used to support the findings of this study are available from the corresponding or the first authors upon reasonable request.

## Abbreviations

ACC: Asini Corii Colla
APH: Acetyl phenylhydrazine
ATP: Adenosine triphosphate
C: Control group
CTX: Cyclophosphamide
DRR: Dry rehmanniae radix
GSH: Glutathione
HCT: Hematocrit
HGB: Hemoglobin
M: Model group
MDA: Malondialdehyde
OPLS_DA: Orthogonal partial least squares discriminant analysis
PCA: Principal component analysis
PLT: Platelet
PRR: Process *rehmanniae radix*
RBC: Red blood cell
RR: *Rehmanniae radix*
SOD: Superoxide dismutase
UPLC-Q-TOF/MS: Ultra-performance liquid chromatography coupled to quadrupole time-of-flight mass
WBC: White blood cell
