# Supplementary material for: Metabolomic strategies and biochemical analysis of the effect of processed Rehmanniae radix extract on a blood-deficient rat model
Source: BMC Complement Med Ther. 2022 Mar 25;22:89. doi: 10.1186/s12906-022-03560-x (PMC8957163; doi:10.1186/s12906-022-03560-x)
Supplement: Supplementary file 1 — Additional file 1. [file 12906_2022_3560_MOESM1_ESM.docx]

**Supplementary materials**

Table 1. The identified potential biomarkers and the changing trend.

| **Metabolites in urine** | **ESI** | **NO.** | **t/R/min** | **m/z** | **Metabolite** | **Molecular formula** | **VIP-value** | **Trend in model, ACC and PRR** |
| --- | --- | --- | --- | --- | --- | --- | --- | --- |
|  | ESI^+^ | 1 | 5.0 | 104.0705 | Dimethylglycine | C_4_H_9_NO_2_ | 3.2 | ↑* ↓****** ↓***** |
|  |  | 2 | 0.7 | 112.0869 | Histamine | C_5_H_9_N_3_ | 3.0 | ↑****** ↓ ↓* |
|  |  | 3 | 1.0 | 114.0662 | Creatinine | C_4_H_7_N_3_O | 20.6 | ↓****** ↓ ↑* |
|  |  | 4 | 1.0 | 116.0706 | L-Proline | C_5_H_9_NO_2_ | 5.2 | ↓***** ↑ ↑* |
|  |  | 5 | 1.0 | 118.0862 | Betaine | C_5_H_11_NO_2_ | 2.9 | ↑* ↓***** ↓***** |
|  |  | 6 | 1.1 | 118.0865 | 5-Aminopentanoic acid / l-valine | C_5_H_11_NO_2_ | 2.9 | ↑* ↓***** ↓***** |
|  |  | 7 | 19.7 | 124.0871 | L-Histidinol | C_6_H_11_N_3_O | 3.5 | ↓***** ↑ ↑* |
|  |  | 8 | 1.0 | 126.0218 | Taurine | C_2_H_7_NO_3_S | 3.3 | ↓****** ↑****** ↑* |
|  |  | 9 | 1.0 | 127.0499 | Imidazoleacetic acid | C_5_H_6_N_2_O_2_ | 3.4 | ↑****** ↓ ↓* |
|  |  | 10 | 1.0 | 132.0766 | Creatine | C_4_H_9_N_3_O_2_ | 9.8 | ↓* ↑****** ↑* |
|  |  | 11 | 0.7 | 137.0709 | 1-Methylnicotinamide | C_7_H_9_N_2_O | 4.3 | ↑* ↑***** ↓* |
|  |  | 12 | 1.0 | 138.0549 | Trigonelline | C_7_H_7_NO_2_ | 3.1 | ↑****** ↓****** ↓****** |
|  |  | 13 | 1.0 | 141.0656 | Methylimidazoleacetic acid | C_6_H_8_N_2_O_2_ | 2.7 | ↓****** ↑****** ↑* |
|  |  | 14 | 1.0 | 144.1018 | L-Carnitine | C_7_H_15_NO_3_ | 4.2 | ↑****** ↓****** ↓****** |
|  |  | 15 | 0.7 | 146.1651 | Spermidine | C_7_H_19_N_3_ | 4.6 | ↓* ↑ ↑***** |
|  |  | 16 | 1.0 | 150.0582 | L-Methionine | C_5_H_11_NO_2_S | 6.5 | ↑****** ↓****** ↓****** |
|  |  | 17 | 1.3 | 166.0860 | L-Phenylalanine | C_9_H_11_NO_2_ | 3.1 | ↑***** ↓****** ↓****** |
|  |  | 18 | 1.0 | 175.0233 | Xanthine | C_5_H_4_N_4_O_2_ | 3.5 | ↓****** ↑****** ↑****** |
|  |  | 19 | 1.0 | 176.0771 | Allantoic acid | C_4_H_8_N_4_O_4_ | 3.0 | ↓****** ↑****** ↑****** |
|  |  | 20 | 5.3 | 180.0657 | 3-Succinoylpyridine | C_9_H_9_NO_3_ | 25.9 | ↑****** ↓****** ↓****** |
|  |  | 21 | 4.0 | 181.0606 | Nicotinuric acid | C_8_H_8_N_2_O_3_ | 3.5 | ↑* ↓****** ↓****** |
|  |  | 22 | 1.0 | 182.0806 | L-Tyrosine | C_9_H_11_NO_3_ | 2.8 | ↑***** ↓ ↓* |
|  |  | 23 | 2.3 | 190.0497 | Kynurenic acid | C_10_H_7_NO_3_ | 3.3 | ↓* ↓****** ↓****** |
|  |  | 24 | 6.3 | 194.0814 | Phenylacetylglycine | C_10_H_11_NO_3_ | 19.8 | ↑****** ↓ ↓* |
|  |  | 25 | 1.3 | 206.0444 | Xanthurenic acid | C_10_H_7_NO_4_ | 2.7 | ↑****** ↓***** ↓****** |
|  |  | 26 | 1.0 | 220.118 | Pantothenic acid | C_9_H_17_NO_5_ | 3.3 | ↑****** ↓****** ↓****** |
|  |  | 27 | 1.0 | 226.0819 | Cytidine | C_9_H_13_N_3_O_5_ | 5.7 | ↑****** ↓****** ↓* |
|  |  | 28 | 1.0 | 242.1134 | Thymidine | C_10_H_14_N_2_O_5_ | 6.5 | ↑****** ↓****** ↓* |
|  |  | 29 | 1.0 | 245.0767 | Uridine | C_9_H_12_N_2_O_6_ | 5.4 | ↓* ↓ ↑* |
|  |  | 30 | 0.7 | 265.1113 | Thiamine | C_12_H_17_N_4_OS | 2.6 | ↑* ↓****** ↓****** |
|  |  | 31 | 5.3 | 287.0999 | Phenylacetylglutamine | C_13_H_16_N_2_O_4_ | 2.6 | ↑****** ↓***** ↓***** |
|  |  | 32 | 16.3 | 480.2791 | Taurocholic acid | C_26_H_45_NO_7_S | 2.7 | ↑****** ↓****** ↓****** |
|  |  | 33 | 1.0 | 96.9602 | Sulfate | H_2_SO_4_ | 19.6 | ↓***** ↑****** ↑****** |
|  |  | 34 | 1.0 | 111.0086 | 2,5-Dioxopentanoate | C_5_H_6_O_4_ | 3.2 | ↑***** ↓****** ↓****** |
|  | ESI**^-^** | 35 | 5.3 | 134.0609 | Dopamine | C_8_H_11_NO_2_ | 4.0 | ↑* ↓****** ↓****** |
|  |  | 36 | 1.0 | 157.0368 | Allantoin | C_4_H_6_N_4_O_3_ | 14.3 | ↑****** ↓****** ↓****** |
|  |  | 37 | 9.7 | 163.0399 | Phenylpyruvic acid | C_9_H_8_O_3_ | 4.1 | ↑* ↓****** ↓* |
|  |  | 38 | 1.0 | 167.0211 | Uric acid | C_5_H_4_N_4_O_3_ | 5.3 | ↑****** ↓****** ↓****** |
|  |  | 39 | 5.3 | 178.0511 | Hippuric acid | C_9_H_9_NO_3_ | 20.2 | ↑* ↓****** ↓****** |
|  |  | 40 | 1.0 | 191.0197 | Citric acid | C_6_H_8_O_7_ | 12.1 | ↓* ↑***** ↑****** |

| **Metabolites in serum** | **ESI** | **NO.** | **t/R/min** | **m/z** | **Metabolite** | **Molecular formula** | **VIP-value** | **Trend in model, ACC and PRR** |
| --- | --- | --- | --- | --- | --- | --- | --- | --- |
|  | ESI^+^ | 1 | 1.0 | 104.1072 | Choline | C_5_H_14_NO | 5.8 | ↓* ↓ ↑* |
|  |  | 2 | 1.0 | 112.0505 | Cytosine | C_4_H_5_N_3_O | 3.5 | ↓****** ↑ ↑***** |
|  |  | 3 | 1.0 | 114.0661 | Creatinine | C_4_H_7_N_3_O | 3.2 | ↑* ↑ ↓* |
|  |  | 4 | 1.0 | 116.0706 | L-Proline | C_5_H_9_NO_2_ | 4.5 | ↑* ↑****** ↓* |
|  |  | 5 | 1.0 | 118.0864 | 5-Aminopentanoic acid | C_5_H_11_NO_2_ | 6.6 | ↓* ↑****** ↑***** |
|  |  | 6 | 1.0 | 133.0971 | D-Ornithine | C_5_H_12_N_2_O_2_ | 3.4 | ↑****** ↑****** ↓****** |
|  |  | 7 | 0.7 | 146.1651 | Spermidine | C_7_H_19_N_3_ | 3.2 | ↑***** ↓ ↓* |
|  |  | 8 | 1.0 | 160.0755 | Indoleacetaldehyde | C_10_H_9_NO | 3.5 | ↑****** ↓****** ↓****** |
|  |  | 9 | 1.0 | 166.0864 | L-Phenylalanine | C_9_H_11_NO_2_ | 9.4 | ↓* ↑ ↑* |
|  |  | 10 | 4.3 | 194.0808 | Phenylacetylglycine | C_10_H_11_NO_3_ | 4.8 | ↑***** ↑ ↓* |
|  |  | 11 | 0.7 | 203.0525 | D-Glucose | C_6_H_12_O_6_ | 5.7 | ↓****** ↑ ↑* |
|  |  | 13 | 8.0 | 300.2891 | Sphingosine | C_18_H_37_NO_2_ | 5.5 | ↑****** ↓****** ↑***** |
|  |  | 13 | 8.3 | 302.3052 | Sphinganine | C_18_H_39_NO_2_ | 5.6 | ↑***** ↓ ↑****** |
|  |  | 14 | 17.0 | 305.2473 | Arachidonic acid | C_20_H_32_O_2_ | 3.2 | ↑****** ↓****** ↓****** |
|  |  | 15 | 9.3 | 400.3420 | L-Palmitoylcarnitine | C_23_H_45_NO_4_ | 3.2 | ↑****** ↓****** ↓***** |
|  |  | 16 | 1.3 | 115.0399 | (R)-2,3-Dihydroxy-isovalerate | C_5_H_10_O_4_ | 3.5 | ↓****** ↑****** ↑****** |
|  | ESI**^-^** | 17 | 1.0 | 128.0351 | Pyroglutamic acid | C_5_H_7_NO_3_ | 7.9 | ↓***** ↑****** ↑****** |
|  |  | 18 | 1.3 | 203.0821 | L-Tryptophan | C_11_H_12_N_2_O_2_ | 3.0 | ↓****** ↓ ↑* |
|  |  | 19 | 8.7 | 378.2403 | Sphimgosine 1-phosphate | C_18_H_38_NO_5_P | 9.0 | ↓* ↑ ↑* |
|  |  | 20 | 6.6 | 514.2823 | Taurocholic acid | C_26_H_45_NO_7_S | 3.0 | ↓****** ↑***** ↑***** |

**Note:** ↑ / ↑ / ↑ represent the up-regulated in model / ACC / PRR, respectively; **↓** / **↓** / **↓** represent the down-regulated in model / ACC / PRR, respectively. *p < 0.05, ** p < 0.01 model group *vs* control group and treatment group *vs* model group respectively.

Table 2. the absorbance value of the different concentration of standard

| Standard concentration/ (mg·ml^-1^) | Absorbance / (A) |
| --- | --- |
| 1 | 3.404 |
| 0.8 | 2.768 |
| 0.6 | 1.990 |
| 0.5 | 1.699 |
| 0.2 | 0.640 |
| 0.1 | 0.210 |

Fig 1. The base peak Chromatogram (BPC)combered between DDR (A) and PPR (B)

Table 3 The difference compound list between DDR and PPR extract based multivariate analysis

| Compound | Formula | Calculated (Da) | Detected (Da) | Error（ppm） | Related changed trend | Time  (min) |
| --- | --- | --- | --- | --- | --- | --- |
| 5-hydroxymethyl furaldehyde | C_6_H_6_O_3_ | 149.0209 | 149.0209 | 0.00 | ↑ | 3.20 |
| Dihydrocatalpol | C_15_H_24_O_10_ | 387.1262 | 387.1259 | -0.77 | ↓ | 1.27 |
| Adenosine | C_10_H_13_N_5_O_4_ | 268.1040 | 268.1035 | -1.86 | ↓ | 1.02 |
| Melittoside | C_21_H_32_O_15_ | 547.1633 | 547.1633 | 0.00 | ↓ | 1.38 |
| Rehmannioside D | C_27_H_42_O_20_ | 709.2161 | 709.2152 | -1.27 | ↓ | 1.26 |
| guaicylglycerol | C_10_H_14_O_5_ | 237.0733 | 237.0753 | -2.11 | **+** | 12.67 |
| Rehmannia glutinin A | C_19_H_34_O_8_ | 413.2146 | 413.2140 | -1.45 | ↓ | 11.26 |
| pterolactam | C_5_H_9_NO_2_ | 116.0706 | 116.0706 | 0.00 | ↓ | 0.97 |
| harman-3-carboxylic acid | C_13_H_10_N_2_O_2_ | 227.0815 | 227.0812 | -1.32 | ↑ | 1.38 |
| Echinacoside | C_35_H_46_O_20_ | 809.2475 | 809.2470 | -0.62 | ↑ | 4.32 |
| Aucubin | C_15_H_22_O_9_ | 369.1156 | 369.1153 | -0.81 | ↓ | 10.65 |
| Leonuride | C_15_H_24_O_9_ | 371.1312 | 371.1308 | -1.08 | - | 2.60 |
| Rehmannia glutinin C | C_19_H_32_O_7_ | 373.2221 | 373.2215 | -1.61 | ↓ | 13.46 |
| Catalpinoside | C_15_H_22_O_10_ | 385.1105 | 385.1102 | -0.78 | ↓ | 14.62 |
| 6-O-Vanilloylajugol | C_23_H_30_O_12_ | 521.1629 | 521.1634 | 0.96 | - | 15.22 |
| Rehmannia glycoside | C_31_H_48_O_18_ | 731.2733 | 731.2728 | -0.68 | ↓ | 17.03 |
